# The stone-to-metal transition reflected in the Iron Age copper production sites of Timna Valley, Israel

**DOI:** 10.1371/journal.pone.0294569

**Published:** 2023-12-19

**Authors:** Ron Shimelmitz, Erez Ben-Yosef

**Affiliations:** 1 Zinman Institute of Archaeology, School of Archaeology and Maritime Civilizations, University of Haifa, Mount Carmel, Haifa, Israel; 2 Department of Archaeology and Ancient Near Eastern Cultures, Tel Aviv University, Tel Aviv, Israel; University of California Santa Cruz, UNITED STATES

## Abstract

Metalwork was a major technological innovation that displaced stone-tool technologies and transformed human society and the environment. However, our understanding of these processes remains partial. In this paper, we approach the stone-to-metal transition from a novel angle–the presence of flint knapping at metal production sites. Drawing on excavations at the Late Bronze and Iron Age copper smelting sites in Timna Valley, Israel, we demonstrate that systematic production of expedient stone tools was integral to these sites’ industrial operations, placing it at the heart of the very same metal circulation networks that were presumably responsible for its displacement. The observations from Timna, coupled with evidence for the use of chipped stone technology in other early Iron Age metallurgical contexts, support the hypothesis that it was probably *both* the high accessibility of iron and its qualities that put an end to the stone tool industry. Copper and bronze could not easily fulfill the function of the *ad hoc* stone tools and were not used to replace stone tools even if they were available and accessible.

## Introduction

The emergence of metalwork constituted a turning point in human history. It provided new technological advantages for humans to shape their environment, displaced chipped-stone tools after ca. three million years, and set in motion a cascade of innovations [1–3]. Nevertheless, the processes that drove the increase in metal tool usage were complex and intertwined, implicating utilitarian advantages, modes of production, circulation networks, and social signaling [4–6]. Thus, while the trajectory from stone to metal was the same throughout, it unfolded along markedly different lines in different regions [5,7,8].

In the Levant, the transition from metal to stone tools was long-drawn-out. Both technologies operated side by side for ca. 4000 years, from the Chalcolithic period to the Iron Age (fifth–first Millennia BC). Stone bifacial tools such as axes and adzes disappeared as early as the Early Bronze Age (ca. 3,700 BC), but sickle blades and expedient flake production (often referred to as *ad hoc*) persisted until the middle of the Iron Age, ninth century BC [6,7,9,10].

Although lithic research of the proto-historical periods often focuses on the more elaborate technologies (e.g., long blade production), it is evident that expedient stone tool production was widely employed by the sedentary communities of the Holocene, constituting one of the most enduring knapped lithic technologies around the globe [11–13]. In England, for instance, Young and Humphery [5] showed that this simple mode of lithic production was associated with domestic activities and lasted into the Iron Age (the first Millennium BC). More importantly, they demonstrated that our limited understanding of the role of lithics in early historical periods is primarily due to poor and inconsistent recovery of such items and a tendency to conceive those found as residual. Similar observations can also be made for the southern Levant. Until recently, *ad hoc* lithic industries were thought to have disappeared at the end of the Bronze Age [10]; now, it is apparent that they lasted well into the Iron Age [6]. Consequently, our understanding of these late lithic industries and how they intertwined with other realms of material culture and technological innovation remains highly fragmented.

One of the striking features of the protracted stone-to-metal displacement is the continuous decrease in flint tools’ symbolic significance. Archaeological and ethnographic research demonstrated that stone tools often constituted active symbolic agents [14–16]. However, as novel technologies like ceramics and metal were introduced in late prehistory, lithics’ symbolic vigor gradually waned [5,17,18]. Thus, by the Chalcolithic period and Early Bronze Age, lithic technology retained only a portion of its original symbolic significance in the Levant [19,20]. Concomitantly, however, as expedient production constituted increasingly greater portions of late prehistoric and early historic lithic assemblages, the strictly utilitarian, non-symbolic significance of these items became the rule [6,17].

Accordingly, scholars have argued that two factors affected the final stages in the transition from stone to metal in the Levant and other regions: (a) function effectiveness and efficiency and (b) access to metal circulation networks [6,7,9,13,21,22]. Functional advantages pertain to the activity conducted, the raw material manipulated, and the specific metal employed [4] and references therein]. Conversely, networks of circulation refer to the relationship between producers and consumers and the availability of metal in daily exchange.

By this token, Manclossi *et al*. [6]: 1284] postulated that the demise of lithic production in the early first-millennium BC Levant was induced by the introduction of iron sickle blades, which provided cutting edges superior to flint (preexisting metals like copper and bronze did not provide the necessary functional advantage). While emphasizing iron’s functional advantage for formal tools like sickles, they also argued that the substitution of *ad hoc* lithic technology, which continued to flourish in domestic settings, depended on metal production and exchange becoming prevalent. Accordingly, stone tools’ utter displacement was only possible once metal became widespread and affordable.

The presence of stone tools in metal production contexts has been reported in previous studies, although these studies mostly focused on ground stones, such as hammerstones and grinding slabs [23–25]. In contrast, the role of knapped stones remained largely unexplored, except for their role in fire igniting equipment [26]; although their presence in some metallurgical context has been noted [27,28]. The new excavations at Timna Valley, Israel, provide a novel access point into the intricacies of lithics’ displacement by metal. The Timna Valley was an industrial region devoted to copper mining and smelting, primarily dated to the late second–early first millennium BC [29,30]. The smelting sites are non-domestic, yet they feature a substantial chipped-stone assemblage. We capitalize on this case to expand the scope of existing discourses on the stone-to-metal transition and do so from within the machinery of metal production. Specifically, we expect that understanding how stone tools were used at the heart of a metal production center will enable us to test the hypothesis that the connectivity and affordability of metals was the primary factor driving the final stages of the stone-to-metal transition.

## Materials and methods

**Fig 1:**
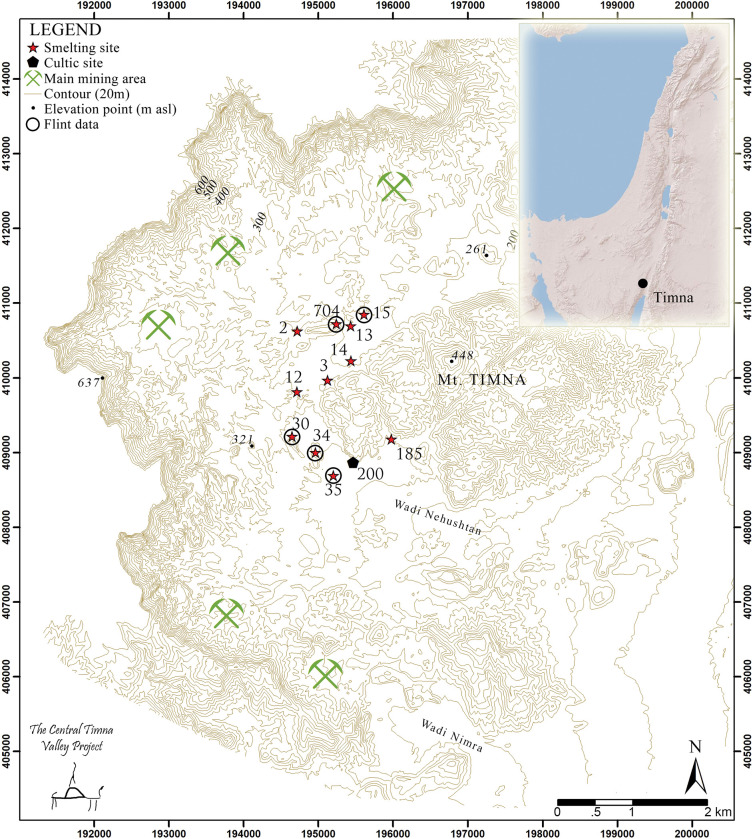
The Timna copper mining and smelting complex dated to the Late Bronze and early Iron Ages (thirteenth–ninth century BC). Sites with lithic assemblages are marked with circles.

Timna Valley is located in the arid region of southern Israel, constituting one of the two main copper production centers of the south Levantine Iron Age. It was investigated between 1959 and 1984 by the Arabah Expedition [29] and has been the focus of a new project since 2012, addressing multiple aspects of early metallurgy and the socio-economic transformations between the Late Bronze and Iron Ages (The Central Timna Valley Project: CTV, [30]) (license provided by Israel Antiquities Authority: G-3, 2013; G-6/2015; G-5/2016; G-5/2017). Timna is composed of dozens of sites engaged in metal mining and smelting (Fig 1). The material culture of these sites is predominantly industrial, indicating various metalworking procedures [31], and it has been suggested that most attendant domestic activities—such as cooking—were conducted in ephemeral tent camps outside of the designated smelting sites [32,33].

**Fig 2:**
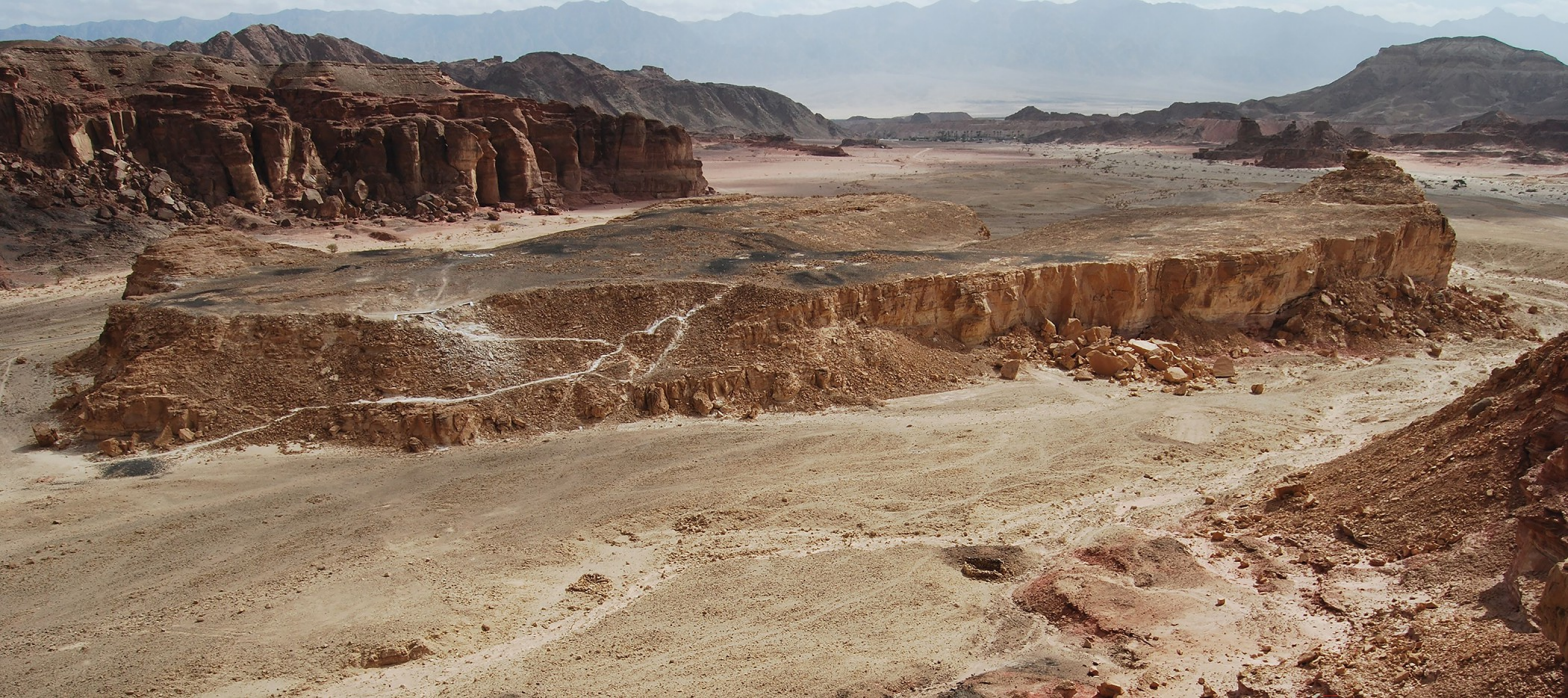
Site 34 (“Slaves’ Hill”), an extensive, early Iron Age copper smelting site on a sandstone mesa.

Over the last decade, the excavation of the main smelting camps of Timna produced a gradually and continuously expanding collection of flint items, which is now too substantial to be explained away as marginal or residual. Thus, we must strive to conceive them as integral to this industrial complex. Towards this goal, we investigate these sites’ lithic assemblages. We mainly draw on Site 34 (“Slaves’ Hill”; Fig 2) and Site 35 (henceforth, T34 and T35), upon which most of the fieldwork focused. We augment these observations with information from Sites 15, 30, and 704 (henceforth, T15, T30, T704). Together, these sites represent continuous copper production in the valley from the thirteenth century BC (Late Bronze Age) to the early/mid-ninth century BC (Iron Age) (Table 1).

**Table 1:** Copper smelting sites in Timna Valley discussed in the paper.

Site	Date	Description	References
T15	Late 12^th^– 11^th^ century BC	An un-walled copper smelting camp (3 acres), located in the upper basin of Wadi Timna. It was excavated in 2015; the excavation focused on several stone features, slag accumulations, and a compound not directly related to the smelting operations.	[30]
T30	Late 12^th^–mid 9^th^ ccentury BC	A copper smelting site in the upper basin of Wadi Nehushtan, surrounded by a wall (~1 acre). The site was excavated by the Aravah Expedition in the 1970s and, in 2009, by one of the authors (EB-Y).	[34,35]
T34	Late 11^th^–late 10^th^ century BC	An extensive copper smelting camp (~7 acres) situated on a sandstone mesa. The mesa has steep cliffs, 20m high on average, and the camp is enclosed by a wall. The site can only be accessed from the north-west. The isolated site was occupied for about a century at the turn of the first millennium BC.	[31,36]
T35	12^th^– 11^th^ century BC	An un-walled, extensive copper smelting camp (~8 acres) located in the upper basin of Wadi Nehusthan. The site was excavated in 2016–2018. The excavation focused on a building complex with several rooms or walled spaces and an attached pen or courtyard.	[30]
T704 (“Crocodile”)	13^th^– 12^th^ century BC	A copper smelting site located on an isolated sandstone cliff. The site was first documented in 2017 as part of the work of the Central Timna Valley Project. It includes an accumulation of slags on the slope and several stone installations.	Unpublished

The lithic finds were retrieved through systematic grid surveys and excavations. The excavations focused on metallurgical installations and piles of industrial waste, most of which were thoroughly sifted with a 1mm mesh and complemented by hand picking to achieve maximum retrieval of small finds [30]. Our analysis has three principal components. First, we seek to determine if and to what extent the lithic artefacts represent a regular and systematic on-site production, which is expected to be reflected through the repeated occurrence of all or most stages of core reduction at the site [12,37]. Second, we explore the assemblages’ compositions, particularly acknowledging the variation within the proto-historical periods, which are characterized by the presence of various *chaînes opératoires* [10]; and third, we engage the possibility that some or all the flint items within the metal production context derive from fractured flint hammerstones. Hammerstones were employed for crushing ores and slags [24,25], operations that often resulted in breakage and accidental flaking [38]. Thus, it is critical to determine if and to what extent flint items are attributable to hammering rather than intentional knapping. We assume the former will be characterized by flint items bearing a high percentage of battered dorsal surfaces and striking platforms (split from hammerstones) alongside a lack of cores and tools.

## Results

### Context and derivation

**Fig 3:**
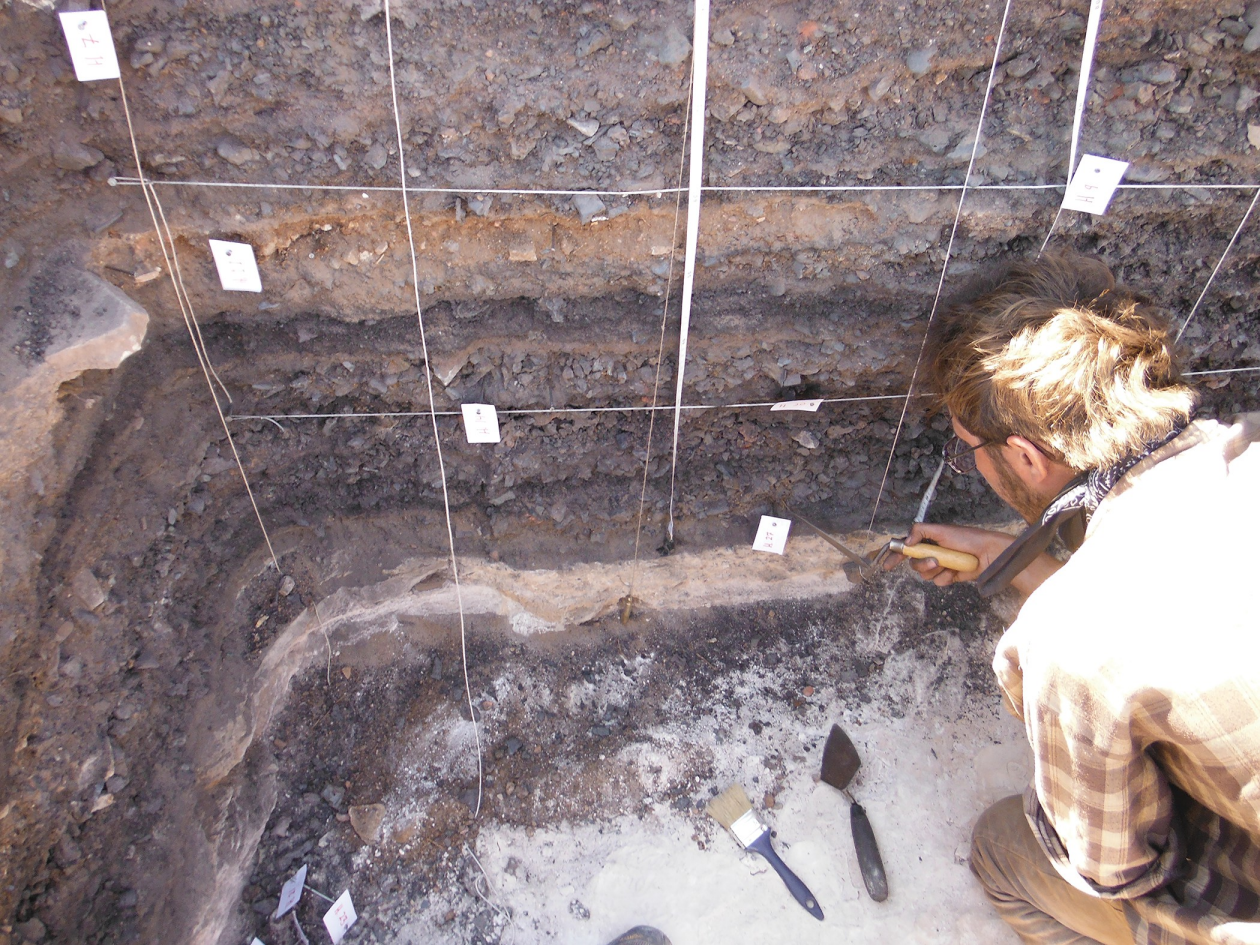
Sampling a section through Slag Mound 19, Site 34 (“Slaves’ Hill”).

All the investigated sites are copper smelting camps consisting of metallurgical installations, storage pits, and substantial amounts of industrial waste. The sediment accumulations vary in breadth and are up to 1m deep (Fig 3), indicating well-organized, systematic large-scale copper processing. All lithic assemblages seem to have been produced on-site. Flint does not naturally outcrop in these sites’ vicinity, which implies that its occurrence is not opportunistic (i.e., low-effort exploitation of an immediately accessible raw material) but calculated. None of the sites includes remains that typologically or technologically predate the Late Bronze Age, overruling the possibility that the flint specimens derive from pre- or protohistoric occupations. Furthermore, T34 enjoys an elevated position on a mesa and features no pre-Iron Age remains [31], indicating that its lithic assemblage is free of external influences and strictly derives from operations conducted at the site.

### Lithic assemblage compositions and technology

The lithics of Timna comprise two large assemblages deriving from Iron Age T34 (n = 796; Table 2) and Late Bronze Age–early Iron Age T35 (n = 536; Table 3) and three small assemblages associated with T15 (n = 53), T30 (n = 30), and T704 (n = 24) (Table 4). The difference in scale reflects the intensity of archaeological work at each of these sites [30]. Given the considerable differences in assemblage size, the following discussion draws primarily on the assemblages of T34 and T35, while the others are only mentioned in passing. In order to evaluate on-site production and the specific stone tool technologies employed, a set of observations were conducted.

**Table 2:** The lithic assemblage of T34.

	*In situ*				Surface				Total					
	Whole	Proximal	Distal	Total	Whole	Proximal	Distal	Total	Whole	Proximal	Distal	Total	% of debitage and tools	% of all assemblage
Primary element flake	5	1	1	7	30	4	3	37	35	5	4	44	12.7	5.5
Primary element blade				0	1			1	1			1	0.3	0.1
Flake	32	12	22	66	95	28	17	140	127	40	39	206	59.5	25.9
Blade	1			1	1			1	2			2	0.6	0.3
Core trimming element	1	1		2	12	1	1	14	13	2	1	16	4.6	2.0
Core				6				16				22	6.4	2.8
Core fragment				6				11				17	4.9	2.1
Tool	6		1	7	19	1	11	31	25	1	12	38	11.0	4.8
Total debitage and tool	45	14	24	95	158	34	32	251	203	48	56	346	100	43.5
Chunk				96				208				304		38.2
Chip				105				3				108		13.6
Raw material				2				4				6		0.8
Hammerstone				4				28				32		4.0
Total	45	14	24	302	158	34	32	494	203	48	56	796		100

**Table 3:** The lithic assemblage of T35.

	In situ				Surface				Total					
	Whole	Proximal	Distal	Total	Whole	Proximal	Distal	Total	Whole	Proximal	Distal	Total	% of debitage and tools	% of all assemblage
Primary element flake	1	1	2	4	6		5	11	7	1	7	15	11.3	2.8
Primary element blade				0				0				0	0.0	0.0
Flake	19	6	4	29	20	9	12	41	39	15	16	70	52.6	13.1
Blade			1	1	1			1	1		1	2	1.5	0.4
Core trimming element			1	1	1			1	1		1	2	1.5	0.4
Core				2				9				11	8.3	2.0
Core fragment				0				7				7	5.3	1.3
Tool	6		2	8	10	1	7	18	16	1	9	26	19.5	4.8
Total debitage and tool	26	7	10	45	38	10	24	88	64	17	34	133	100	24.8
Chunk				127				208				335		62.5
Chip				50				3				53		9.9
Raw material								5				5		0.9
Hammerstone								10				10		1.9
Total				222				314				536		100

**Table 4:** The lithic assemblages of T15, T30, and T704.

	Site T15				Site T30				Site T704			
	Whole	Proximal	Distal	Total	Whole	Proximal	Distal	Total	Whole	Proximal	Distal	Total
Primary element flake	1			1	1		1	2				0
Primary element blade				0				0				0
Flake	5	2	2	9	2		2	4	7			7
Blade	1			1				0				0
Core trimming element	1			1	1			1	1			1
Core				0				2				4
Core fragment				0				2				0
Tool	6			6	1			1	5		2	7
**Total debitage and tool**	**14**	**2**	**2**	**18**	**5**	**0**	**3**	**12**	**13**	**0**	**2**	**19**
Chunk				20				15				3
Chip				12				1				1
Raw material				1				0				0
Hammerstone				2				2				1
**Total**	**14**	**2**	**2**	**53**	**5**	**0**	**3**	**30**	**13**	**0**	**2**	**24**

The raw material is diverse and mostly of medium to low quality. A small group of 27 items made on white-pink flint and a single hematite flake are notable. Interestingly, while in T35, 62.5% of the cores and 42.9% of the blanks and tools had a calcareous cortex, suggesting primary geological derivation, in T34, only 6.7% of the cores and 25.4% of the blanks and tools had comparable cortexes, implying the procurement of rolled and patinated items typical of secondary geological sources (Table 5; for correlation between neo-cortex and primary and secondary geological sources, see [39,40]).

**Table 5:** The distribution of cortex types on cores and knapped items (shaped and unshaped).

	Calcareous	Patinated	Rolled	Total	n
Cores					
Site T34	6.7	46.7	46.7	100	15
Site T35	62.5	37.5	0.0	100	8
blanks and tools					
Site T34	25.4	43.2	31.4	100	118
Site T35	42.9	28.6	28.6	100	49

**Fig 4:**
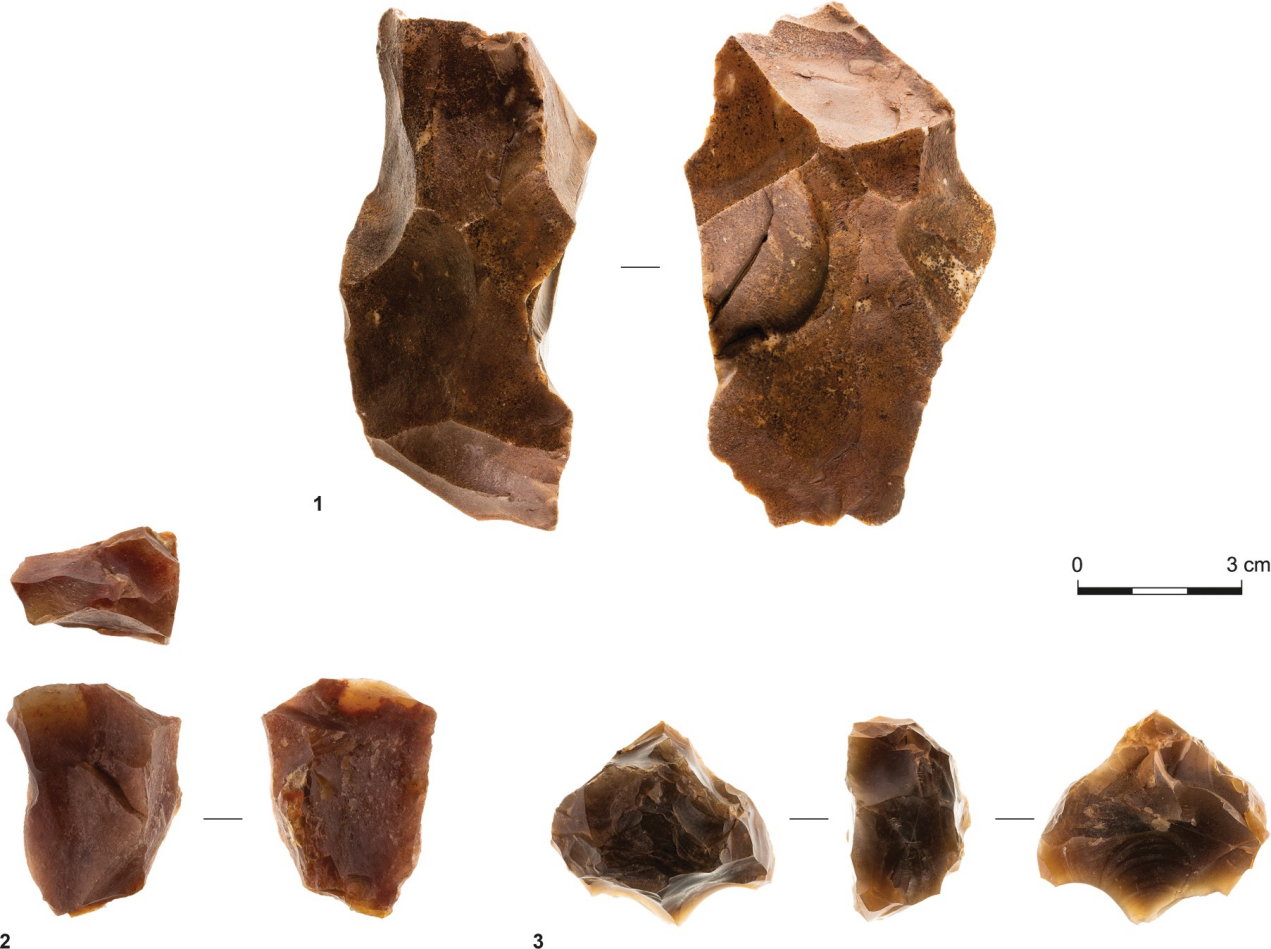
Multi-striking platform cores; observe that no. 1 is double-patinated.

Sites T34 and T35 have similar assemblage compositions. Flakes constitute 59.5% and 52.6% of their debitage and tools, respectively, and blades are scarce, accounting for 0.6% and 1.5% of the respective assemblages. Furthermore, cores and core fragments are well represented, indicating on-site flint knapping. In T34, they comprise six single-striking platform cores and 16 multi-striking platform cores, while in T35, they consist of multi-striking platform cores only (Fig 4). Preliminary shaping of cores was not observed, and reduction seems to have been simple.

Tables 6 and 7 describe the characteristics of the flakes’ striking platforms and end terminations. Investment in striking platform preparation is primarily reflected in the presence of dihedral, faceted, and multi-scared platforms (accounting for 25.1% and 19.7% of the flakes of T34 and T35, respectively). Nonetheless, the presence of natural or battered striking platforms (18.6% and 12.1% of the flakes of T34 and T35, respectively), which indicate the lack of preliminary preparation, is also of note. Table 8 presents the items’ metrics, underscoring the blanks’ small size (ca. 3cm long).

**Table 6:** Striking platforms of primary element flakes and flakes (blanks and shaped).

	Large plan	Thin plan	Punctiform	Faceted	Dihedral	Multi-scarred	Natural	Battered	Total	n
Site T34	47.5	7.7	1.1	12.0	9.3	3.8	11.5	7.1	100	183
Site T35	53.0	15.2	0.0	7.6	9.1	3.0	12.1	0.0	100	66

**Table 7:** End termination of primary element flakes and flakes (blanks and shaped).

	Feathered	Hinged	Overshot	Total	n
Site T34	76.2	18.9	4.9	100	185
Site T35	72.5	10.1	17.4	100	69

**Table 8:** Metrics (mm) of primary element flakes (PE flakes) and flakes (blanks and shaped; whole items only).

		Length (mm)		Width (mm)		Thickness (mm)		n
		Mean	s.d.	mean	s.d.	mean	s.d.	
Site T34	Flake	29.6	11.3	27.8	9.1	8.5	4.7	137
	PE flake	33.1	10.2	29.5	10.6	10.2	4.1	41
	Total	30.4	11.2	28.2	9.5	8.9	4.6	178
Site T35	Flake	29.0	9.1	25.7	7.7	8.6	3.4	46
	PE flake	46.2	10.5	40.0	8.7	16.2	4.9	14
	Total	32.5	11.6	28.9	9.9	10.4	4.9	60

T34 and T35’s core trimming elements are irregular, and none is an overshot, core tablet, or crested blade. Alongside the cores’ character, their irregularity suggests that they are unlikely to have derived from preplanned core pre-shaping or rejuvenation actions. Instead, they probably resulted from the exploitation of multi-striking platform cores. Altogether, the evidence portrays a regular exploitation of expedient, simple flake, production at the site and the lack of any other reduction sequence or its products (such as sickle blades or other specific tools that occurred in the Late Bronze and the Iron ages [10,41]).

**Fig 5:**
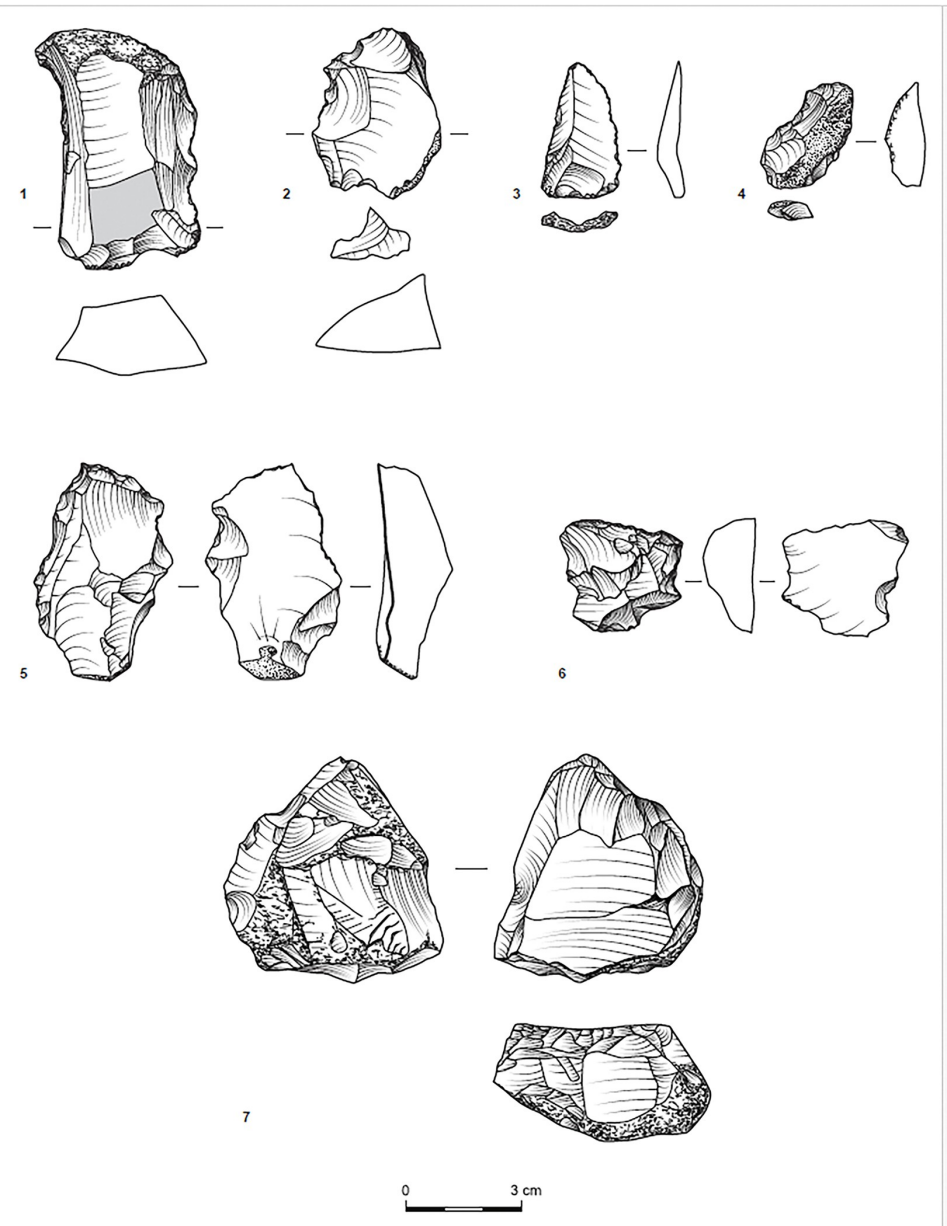
Denticulates (1, 2, 5), a retouched flake (3), scrapers (4, 6), and a core (7), Site T35.

**Fig 6:**
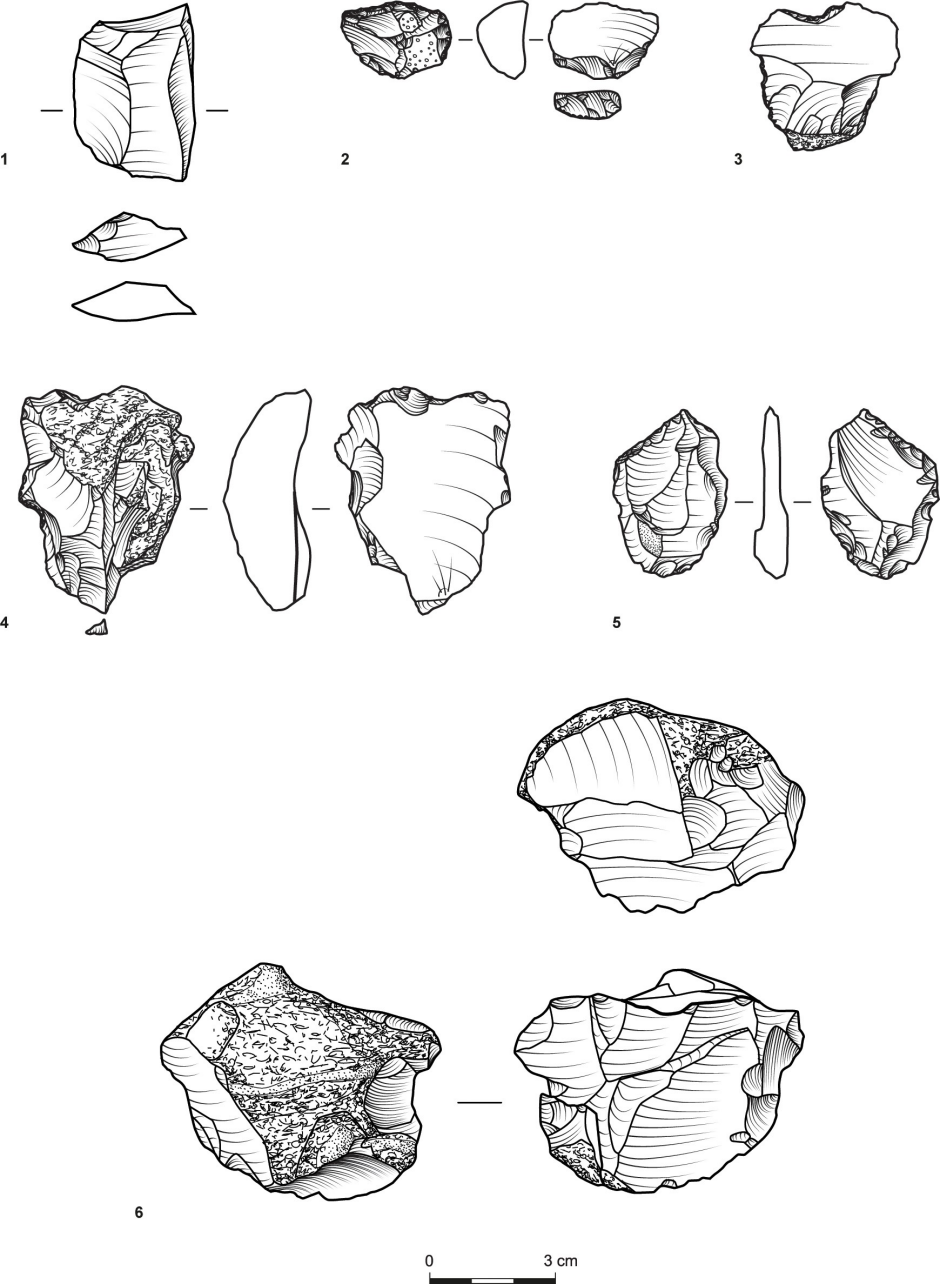
A flake (1), a scraper (2), notch/denticulates (3, 4), a retouched item (5), and a core (6), (1–5: Site T35; 6: Site T34). Items 1, 4, 6 bear crushed surfaces.

**Fig 7:**
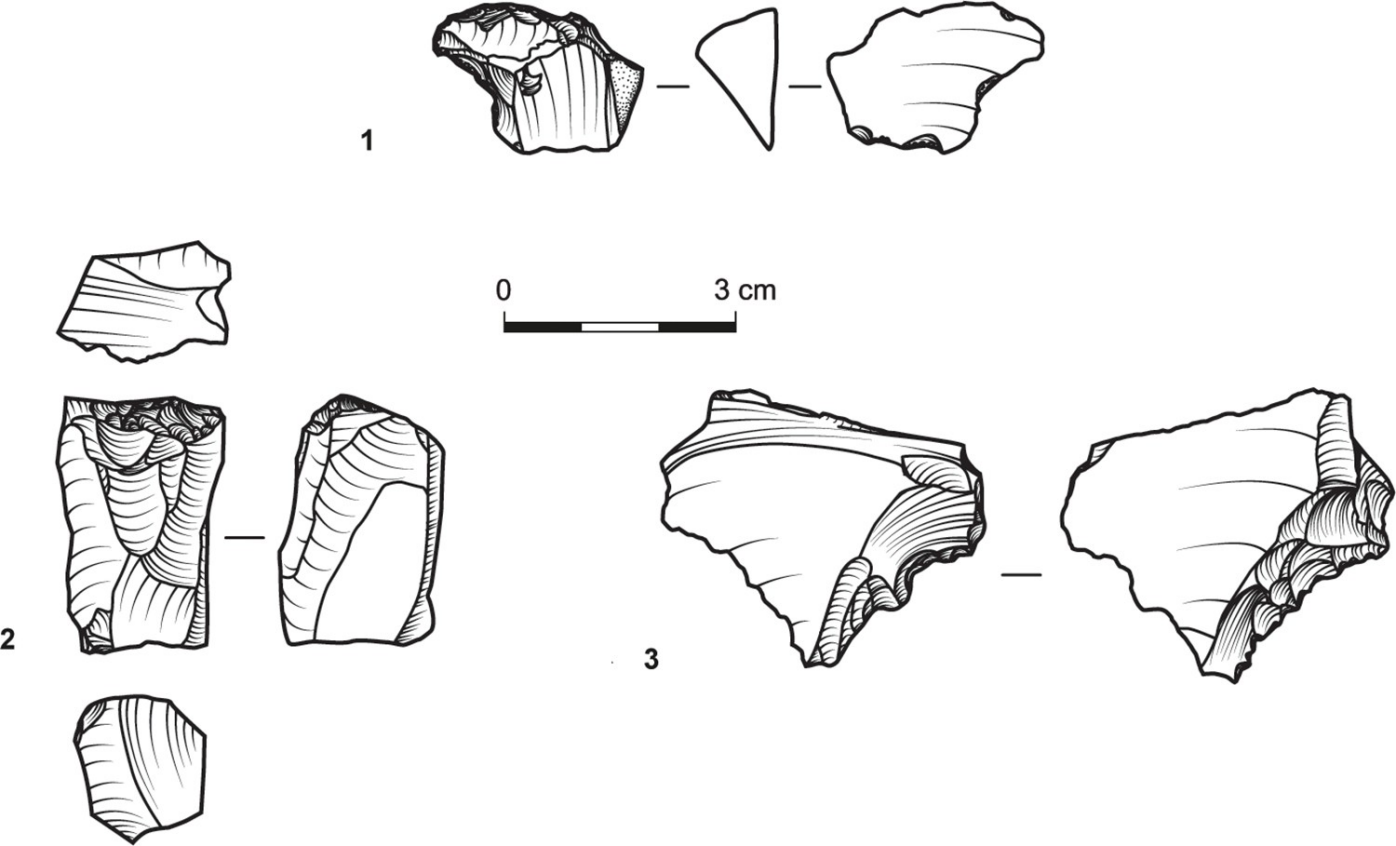
A scraper (1; Site T15), a core (2; Site T30), and an awl (3; Site T704).

**Fig 8:**
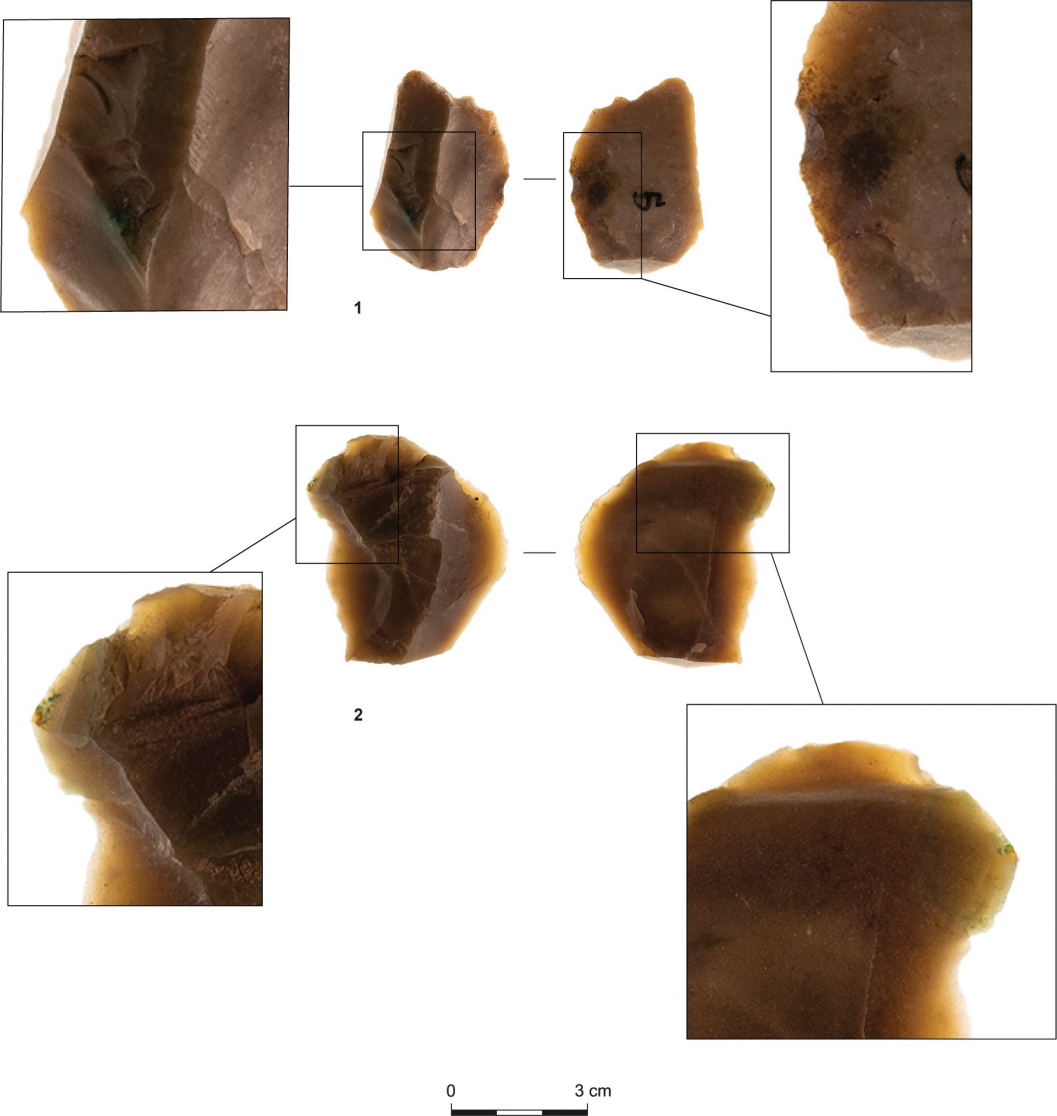
Flakes bearing green mineral residue.

The tools (Table 9) were shaped on relatively large blanks (Table 10), a pattern also noted for other contemporary assemblages [41]. They primarily include retouched flakes and denticulates/notches (Fig 5:1, 2, 5, Fig 6:3, 4), which further support the expedient character of the assemblages. Nonetheless, the scarcity of awls (Fig 7:3) and the notable occurrence of scrapers in T34 and T35 (Fig 5:4, 6, Fig 6:2, Fig 7:1) suggest the use of chipped stones was not restricted to cutting edges. While the stone tools’ specific applications remain indeterminate, three flakes bear traces of a green mineral, probably copper ore (Fig 8).

**Table 9:** The flint tools per site.

	Awl	Denticulate/notch	Retouched flake	Scraper	Unidentified tool fragment	Total
Site T704 (n)	1	2	2		2	7
Site T15 (n)		1	5			6
Site T30 (n)			1			1
Site T34 (n)		11	18	7	2	38
Site T35 (n)	1	8	15	2		26
Site T34 (%)		28.9	47.4	18.4	5.3	100
Site T35 (%)	3.8	30.8	57.7	7.7		100

**Table 10:** The mean measurements of flakes, addressing both blanks and shaped items (measurements deformed by extensive retouching are excluded); the difference between blank and tool thickness is statistically significant: t(181) = -3.131, p = 0.02.

	Length			Width			Thickness		
	n	Mean	s.d	n	Mean	s.d	n	Mean	s.d
Blanks	158	29.2	11.2	156	26.9	8.7	160	8.1	4.1
Tools	16	31.2	8.4	19	29.8	9.6	23	11.0	4.6

### Hammerstone breakage

Lastly, let us explore the relationship between the hammerstones and the knapped material. Altogether, 47 flint hammerstones were found in Timna: 32 in T34 (4.0%), 10 in T35 (1.9%), and five in T15, T30, and T704 (4.7% in all three together). They are made of various flint types, but none is of the white-pink raw material noted above. Only seven are whole, ranging in maximum size between 6.7cm and 11.4cm, measuring on average 8.1cm (s.d. 1.5cm). Seven of the fragmented hammerstones are represented by halves, while the remaining 33 are comprised of smaller pieces.

**Fig 9:**
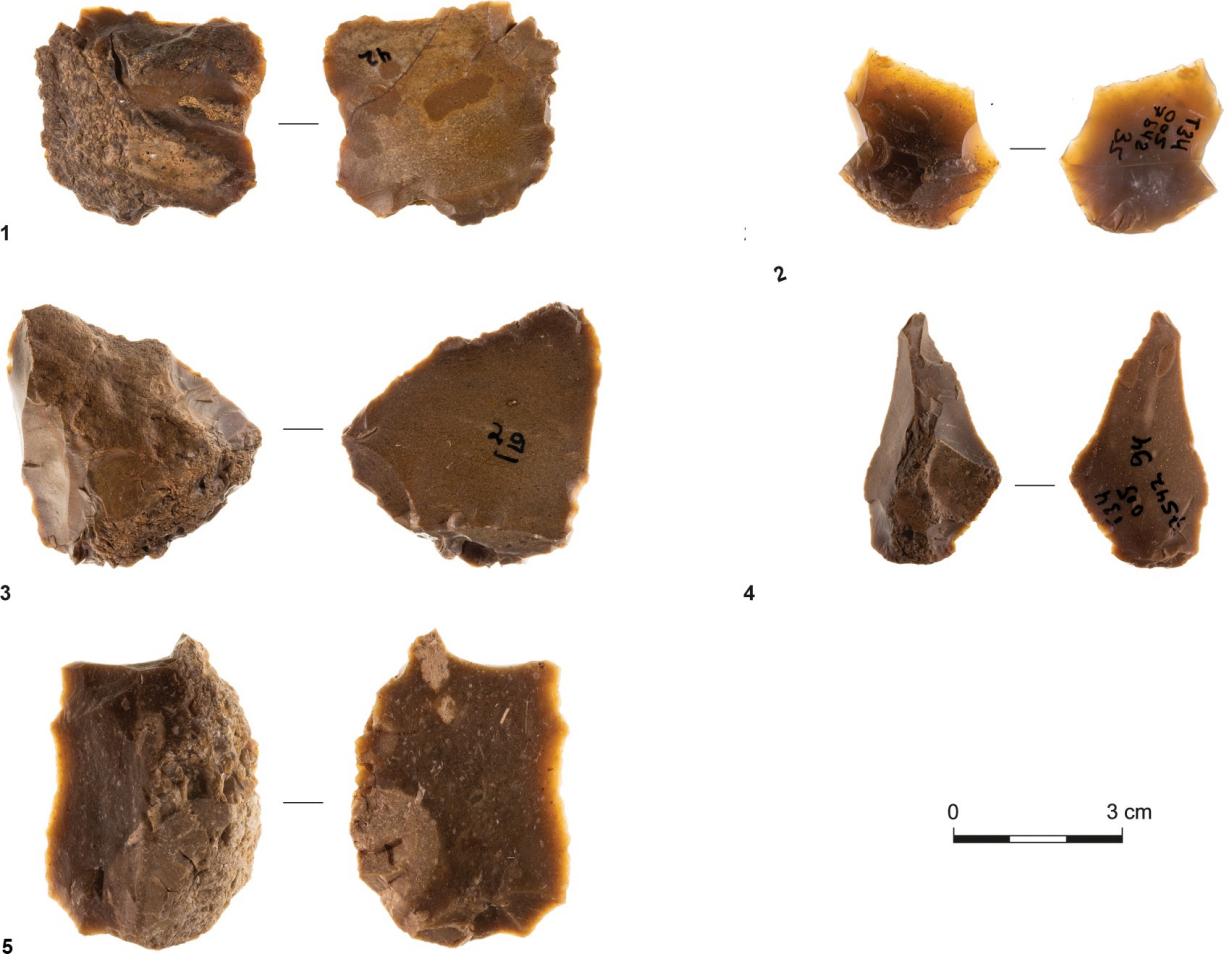
Flakes detached from hammerstones.

Among the debitage, particularly pertinent are flakes and primary element flakes with battered surfaces predating their detachment (T34, n = 28; T35, n = 8; T704, n = 2; Fig 9). In nine, only the striking platform is battered (Fig 5:3); in eight, the battering extends across part or all of the striking platform and the dorsal face; and in 11 it spreads across the dorsal face only. Five of the flakes bearing battering marks were modified into tools (Fig 5:1; Fig 6:3, 4). Proportionally, however, these items comprise only a fraction of the flakes and primary element flakes (including both blanks and tools) in sites T34 (9.72%) and T35 (7.3%).

An effort to single out hammerstones from which flint items were detached produced only five items. One (retrieved from T30) is a hammerstone with a single conchoidal scar, which could have been induced while pounding. The other four (T34, n = 3; T704, n = 1) are cores bearing a combination of extensive battered surfaces and flake removals. These removals undoubtedly originated from striking platforms, indicating that these hammerstones were purposefully recycled into cores (Fig 5:7, Fig 6:6).

Altogether, given the low number of items bearing battered surfaces and that most cores—the principal indicator of on-site production—cannot be traced back to hammerstones, the extent and regularity of this practice must be marginal in the creation of the assemblage.

## Discussion and conclusions

Timna is exempt from one of the most significant hurdles to the systematic analysis of historical-period lithic industries: the high portion of pre- and protohistoric residual materials [10]. Not even one item in the Timna assemblages was identified to be technologically or typologically earlier to the sites, and no indications of intrusive materials were observed. Thus, we can confidently consider the flint artefacts analyzed above integral to the Late Bronze and Iron Age smelting sites.

More specifically, the technological analysis highlights the presence of cores, core trimming elements, primary element flakes, flakes, and a small range of tools, which unequivocally indicate on-site lithic production. Furthermore, the narrow range of flake sizes (Table 8) and the selection of relatively large blanks for tool shaping (Table 10) indicate that flint reduction was calculated and systematic. This, however, is not to say that it was constant throughout the sites’ operations. Raw material procurement strategies shifted from a preference for primary geological sources in the twelfth–eleventh centuries BC (Site T35) to an almost exclusive reliance on secondary geological sources in the tenth century BC (Site T34). In turn, this shift speaks for technological negotiations at the very end of chipped stone tools’ historical trajectory and a reduced attention to the quality of raw material procured.

Concomitantly, it is also evident that some portions of the flint assemblage derive from recycled and fragmented flint hammerstones [24,42], best demonstrated by flakes with battered striking platforms (Table 6; Fig 5:1; Fig 6:3). Notably, the recycling of hammerstones into cores implicates low-quality products due to internal fractures caused by repeated pounding. However, insofar as flint production at Timna was primarily expedient and aimed for sharp edges, this is unlikely to have been of much consequence. Moreover, the scale of this phenomenon was evidently rather small (less than 10% of the flakes and primary element flakes) and thus cannot be regarded as the assemblages’ main reduction sequence.

Given these observations, what can the Timna lithic assemblages teach us about the mechanisms behind the transition from stone tools to metal? The discussed assemblages derive from industrial contexts and seem to have participated in the copper processing operations, as implied from their contexts and by the lithic items bearing copper ore residue (Fig 8). Significantly, stone tools, in general, and flint implements, in particular, have been observed in primary (smelting) and secondary (recycling, tool production) metallurgical contexts across the Mediterranean basin and beyond. These cases primarily include ground stone tools, mostly hammerstones and grinding stones, which have been systematically described in a set of papers [e.g. [24,42–47]. However, some feature knapped flint, either as implements in fire-ignition equipment (e.g., the eighth–seventeenth-century AD Indonesian iron production sites; [26]) or as retouched tools and debitage that were probably used for cutting and scraping. In the Mediterranean basin, the latter chiefly occur in third and second-millennia BC copper smelting sites and bronze workshops [47–49]. Significantly, several instances of flint tools in bronze workshops of the early Iron Age—just before systematic flint knapping disappeared forever from the East Mediterranean—have been reported as well (e.g., Tel Rehov and Tel Dan; [27,28,50]. Nonetheless, it is the robust body of evidence from Timna that best confirms that expedient flint production was integral part in Iron Age metal processing.

The regular and organized use of knapped flint tools within metallurgical contexts undermines the prevalent understanding that the contraction of the flint knapping industries to expedient production entailed their withdrawal from non-domestic settings in the Levant [6,10] and elsewhere [13,22]. Moreover, the use of flint in contexts where metal was readily available–the metal workshop–calls for a reevaluation of some aspects of the stone-to-metal transition. Most notably, they call for reconsidering tool utility and communities’ integration in metal circulation networks as determining factors driving the transition to a close in the Levant [6]. The superiority of metal active edges has long been recognized as such factor. In the Levant, it is manifested in a millennia-long replacement of lithic implements, first comprising woodworking tools (e.g., axes and adzes), then arrowheads, and finally sickle blades. Against these displacements, *ad hoc* stone tools proved to be the most resilient [6,7,10]. One should ask, accordingly, why these simple tools were not replaced earlier. How come they persisted for so long in domestic and industrial contexts, including those related to metal (copper and bronze) production?

As *ad hoc* flint implements are functionally diverse, opportunistic, and contingent, no experimental program can be designed to fully compare the efficacies of iron, bronze, copper, and flint. Previous research emphasized that the replacement of this simple industry unfolded gradually from the onset of the Middle Bronze Age and culminated once metal had become affordable [6]. We can, however, approach it from a different angle and ask whether *ad hoc* stone tools, which form the majority of stone tools of the discussed periods, flourished before the introduction of iron because other metals were relatively expensive and inaccessible or because they were unsuitable for the tasks at hand. The mines in Timna, which extracted copper, not iron, are precisely the platform to better answer this question. Our observations span the periods in which iron gradually became widespread in the southern Levant, eventually replacing copper/bronze in dominance during the late tenth and ninth century BC [51]. If access to metal, in general, was the main issue, there would have been no reason to find a substantial amount of flint tools at Timna, a hub of copper circulation. Therefore, it is more likely that the relatively soft copper and bronze, which characterized Timna and the other aforementioned Levantine metallurgical contexts, were unfitting for all tasks performed by expedient flint tools. Thus, while iron clearly brought the stone-to-metal transition to a close thanks to its widespread distribution, affordability and hard active edges, our analysis of the Timna’s flint assemblages underscores that the combination of these qualities provided iron with the impetus to tip the balance and that one without the other would not have led to the complete replacement of stone tools.

## Supporting information

S1 Data (XLSX)
